# Hospital admissions from a pediatric HIV care and treatment program in Malawi

**DOI:** 10.1186/s12887-016-0556-3

**Published:** 2016-01-30

**Authors:** Carl A. Nosek, W. Chris Buck, Alison C. Caviness, Abbie Foust, Yewo Nyondo, Madalitso Bottomani, Peter N. Kazembe

**Affiliations:** Baylor Children’s Foundation Malawi, Lilongwe, Malawi; Department of Pediatrics, University of California San Francisco, San Francisco, California USA; Department of Pediatrics, University of California Los Angeles, Maputo, Mozambique; Department of Pediatrics, Baylor College of Medicine, Houston, Texas USA; Department of Pediatrics, University of Colorado, Aurora, Colorado USA

**Keywords:** HIV, Pediatric, Admission, Hospitalization

## Abstract

**Background:**

The scale up of pediatric antiretroviral treatment programs across Sub-Saharan Africa over the last decade has brought increasing numbers of children into HIV care. This patient population requiring life-long care presents new challenges in the outpatient and inpatient settings. We sought to describe hospitalizations from a large pediatric HIV treatment facility to better understand the scope of the situation and identify areas for improved care delivery.

**Methods:**

We conducted a retrospective case series of all HIV-infected and exposed patients <18 years enrolled at Baylor College of Medicine Children’s Foundation Malawi, from October 2004-October 2010. Patients admitted to the hospital on or after the day of enrollment were included. Data were extracted from electronic clinic records. Analysis was done at the patient and admission level, as some patients had multiple admissions.

**Results:**

Of 5062 patients enrolled in care, 877 (17.3 %) had 1137 admissions at median age 24 months (IQR: 12–62). 191 (21.8 %) patients had multiple admissions. A high proportion of admissions occurred in patients under two years (49.4 %), those within one month of clinic enrollment (32.9 %), those with severe immune suppression (44.0 %), and those not on ART (48.5 %). The frequency of primary admission diagnoses varied across these same variables, with malnutrition, pneumonia, and malaria being the most common.

**Conclusions:**

Illness requiring hospitalization is common in HIV-infected and exposed children and these results reinforce the need for a comprehensive care package with special attention to nutrition. Strengthened programs for malaria prevention and expanded access to pneumococcal vaccine are also needed. The high burden of admissions in children under 24 months and those newly enrolled in care suggests a need for continued improvement of early infant diagnosis and provider-initiated testing programs to link patients to care before they are symptomatic. Similarly, the high proportion of admissions in those not yet started on ART emphasizes the importance of rapid initiation of ART for eligible pediatric patients.

## Background

Sub-Saharan Africa has the highest burden of pediatric HIV in the world, with over 90 % of the estimated 3.2 million children living with HIV globally. The last decade has seen a massive scale-up of antiretroviral treatment (ART) programs in the region, and 551,065 children were reported to be receiving ART in sub-Saharan Africa at the end of 2012 [1]. Supporting these huge numbers of patients on life-long ART is increasingly stressing already weak national health systems, and the challenges in the chronic outpatient management of HIV are well-documented [2, 3].

Much less has been published about the impact of the pediatric HIV epidemic on inpatient facilities that care for children. Studies done prior to the roll-out of national ART and prevention of mother to child transmission (PMTCT) programs reported very high HIV prevalence in admitted children with corresponding high inpatient mortality rates (29 and 17 %, respectively in one study from Soweto in 2000) [4–9]. More recent research has demonstrated decreasing, but still overall high HIV prevalence and associated mortality in pediatric admissions (19 and 12 %, respectively in a follow-up study from the same hospital in Soweto in 2010) [10, 11].

Despite this progress, regional pediatric ART coverage rates are still very low (only 32 % of eligible infected children were on treatment in 2012), and much of the inpatient HIV burden likely still comes from children not yet enrolled in care and treatment [1]. However, as national ART programs continue to expand and mature, and vertical transmission rates continue to decrease, it is probable that a larger percentage of hospitalized HIV-infected children will already be enrolled in HIV care. There is very little published literature on this subject using the outpatient HIV care clinic as the starting point for analysis, rather than the pediatric ward [12].

In this context, we conducted a retrospective review of hospital admissions from a large cohort of children receiving HIV care in Malawi, seeking to characterize this specific patient population and better understand the implications for both the inpatient and outpatient settings.

## Methods

This was a retrospective case series of all children (<18 years) who were enrolled in care at the Baylor College of Medicine-Abbott Fund Children’s Clinical Centre of Excellence (COE) between October 2004 and October 2010 and had a documented hospital admission in their outpatient records. The COE provides comprehensive HIV care (including TB treatment, supplemental and therapeutic foods for malnutrition, and sick visits) to patients in the Lilongwe area, serves as a national HIV referral center, and is the largest provider of pediatric ART in Malawi with approximately 8 % of all children on treatment at the time of this analysis [13]. The COE, an outpatient facility, is located immediately adjacent to Kamuzu Central Hospital (KCH), the regional referral hospital, which has about 14,000 pediatric admissions per year and a previously reported pediatric inpatient HIV-infection and exposure prevalence of 8.5 and 6.5 % respectively [11, 14]. There is a strong referral system between the COE and KCH, both for admissions from clinic as well as referrals for outpatient HIV care for those newly diagnosed on the wards. Clinicians from the COE also provide HIV consultative services on the KCH pediatric wards.

All study data came from the COE’s outpatient electronic medical record (EMR). Paper-based inpatient KCH records were not used to supplement the information contained in the EMR, as it was not logistically feasible to link them to clinic files. Patients with a documented admission on or after the day of registration in HIV care at the COE were eligible for inclusion. All HIV-infected children were eligible for inclusion. Patients who were enrolled as exposed and later tested definitely HIV-infected were also included, as were infants who were still considered exposed without definitive HIV testing as of Oct. 2010. The only patients who were excluded based on HIV status were those who enrolled as exposed, but were later discharged as definitively HIV-uninfected. All patients enrolled in the COE have electronic medical records and no patients were excluded for lack of records.

A list of patients with documented admissions was generated by an EMR query. Patient EMR records for each admission (including post-hospital discharge records when available) were then manually reviewed to collect study data including primary admission diagnosis and other secondary admission diagnoses. Acute nutritional status (per Malawi guidelines using weight/length, mean upper arm circumference (MUAC,) and edema assessments), TB status, WHO staging, and CD4 closest in time to admission (+/− 6 months from admission date) were also recorded [15]. These data were entered into an Access® database (Microsoft Corporation, Redmond, WA, USA) and merged with other clinical and demographic data extractable from the EMR without chart review.

Data analysis was performed using SPSS 20.0 (IBM, Chicago, IL) and was primarily descriptive. Continuous variables were categorized for further description using frequencies. Patient age was categorized as 0–11, 12–23, 24–35, 36–59, 60–119, 120–179, greater than and equal to 180 months. Patient age was also described using medians and interquartile ranges. CD4 results were stratified according to WHO age-based immune classifications, with preferential use of CD4% in children less than 5 years, and absolute CD4 in those greater than and equal to 5 years [16]. Time since enrollment was categorized into first visit, 1–30, 31–90, 90–365, and greater than 365 days. Time on ART was categorized into none, less than 1, 1–2, 3–6, 7–12, and greater than 12 months. All other patient and admission-level variables were categorical and described using frequencies.

Analysis was done primarily using admissions as the base variable since some patients had more than one admission and variables such as ART status, age, immune status, etc. varied from one admission to another. Also, since many admissions were associated with multiple diagnoses, the frequency of admission diagnoses was described separately as primary (as identified from the EMR) and overall (any diagnosis for the admission, independent of whether it was primary). Stacked bar graphs were used to graphically describe the frequency of primary admission diagnoses within categories of patient age, time from enrollment in HIV care, immune status, and time from ART initiation.

This study was conducted as part of a general retrospective EMR review protocol that was approved by both the Baylor College of Medicine and Institutional Review Board and the Malawi National Health Sciences Research Committee. Under these ethics approvals, informed consent was not required for retrospective analysis of de-identified routine clinical care data.

## Results

A total of 877 individual patients (median age 24 months (IQR: 12–62), 46.8 % female, 53.2 % male) were identified as having 1137 separate admissions, representing approximately 17.3 % (877/5062) of the comparable clinic population ever enrolled. The large majority of patients (685/78.1 %) only had one documented admission, while multiple hospitalizations were documented for 192 (21.9 %) patients, and 19 (2.2 %) had four or more admissions. Patients with an oncologic diagnosis on their first admission were more likely to be admitted more than one time (OR 6.1, 95 %CI 1.4-25.7) over the duration of the study. No other first-admission variable (age, WHO stage, immune status, nutritional status, ART, TB, or diagnosis) was significantly associated with multiple admissions.

Among this group of admitted patients, 393 (44.8 %) were alive and in care at the end of the study period, 271 (30.9 %) had died (not necessarily during the hospital admission), 119 (13.6 %) had transferred out to other facilities/outpatient clinics, and 94 (10.8 %) were lost to follow up as of October 2010. Additional patient-level data is found in Table 1.

**Table 1:** Characteristics of 877 patients admitted to the Hospital, Malawi 2004–2010

	Number	Percent
Sex		
Female	410	46.8
Male	467	53.2
Number of Admissions		
1	685	78.1
2	152	17.3
3	21	2.4
4–8	19	2.2
Patient Status as of October 2010		
Active	393	44.8
Died	270	30.8
Lost to follow-up	95	10.8
Transferred out	119	13.6
Age at First Admission		
≤ 11 months	218	24.9
12–23 months	229	26.1
24–35 months	106	12.1
36–59 months	101	11.5
60–119 months	117	13.3
120–179 months	78	8.9
> 180 months	28	3.2
Time from Clinic Enrollment to First Admission		
First Visit	208	23.7
1–30 days	157	17.9
31–90 days	163	18.6
90–365 days	207	23.6
> 365 days	142	16.2

Analysis of admission-level data revealed that almost half (562, 49.4 %) of the total 1137 admissions occurred in patients under two years of age. Related to the time from clinic enrollment, 207 (18.2 %) occurred on the child’s first visit and 374 (32.9 %) occurred within one month. Of the 551 admissions in HIV-infected patients not yet on ART, 216 (39.2 %) were enrolled in the previous 14 days and 286 (51.9 %) in the previous 30 days.

Most admissions were in patients with advanced clinical staging (934, 82.1 % were WHO III or IV.) Severe suppression was the most common immune status at admission (501, 44.0 %), however 230 (20.2 %) of admissions were in patients with no immune suppression.

Many admissions were associated with multiple diagnoses—there were 2951 total diagnoses identified for the 1137 admissions. Malnutrition and pneumonia were the most common primary diagnoses (26.6 and 18.6 %, respectively) and overall diagnoses (13.6 and 13.6 % respectively (Table 2).

**Table 2:** Frequency of primary and overall diagnoses for 1137 admissions, Malawi 2004–2010

	Primary diagnosis N (%)	All diagnoses N (%)
Malnutrition	302 (26.6 %)	401 (13.6 %)
Pneumonia	212 (18.6 %)	402 (13.6 %)
Malaria	112 (9.9 %)	233 (7.9 %)
Gastroenteritis	68 (6.0 %)	294 (10.0 %)
Sepsis	68 (6.0 %)	330 (11.2 %)
Other Infection	53 (4.7 %)	86 (2.9 %)
PCP Pneumonia	48 (4.2 %)	74 (2.5 %)
Tuberculosis	45 (4.0 %)	150 (5.1 %)
Kaposi Sarcoma	43 (3.8 %)	66 (2.2 %)
Anemia	38 (3.3 %)	174 (5.9 %)
Neurologic	28 (2.5 %)	78 (2.6 %)
Oncologic	19 (1.7 %)	21 (0.7 %)
Pulmonary (including LIP)	16 (1.4 %)	38 (1.3 %)
Medication Adverse Effect	15 (1.3 %)	18 (0.6 %)
Candidiasis	8 (0.7 %)	393 (13.3 %)
Hematologic	5 (0.4 %)	78 (2.6 %)
Cryptococcal Meningitis	4 (0.4 %)	5 (0.2 %)
Other	36 (3.2 %)	95 (3.2 %)
Unknown	17 (1.5 %)	17 (0.6 %)
TOTAL	1137 (100 %)	2951 (100 %)

**Fig. 1:**
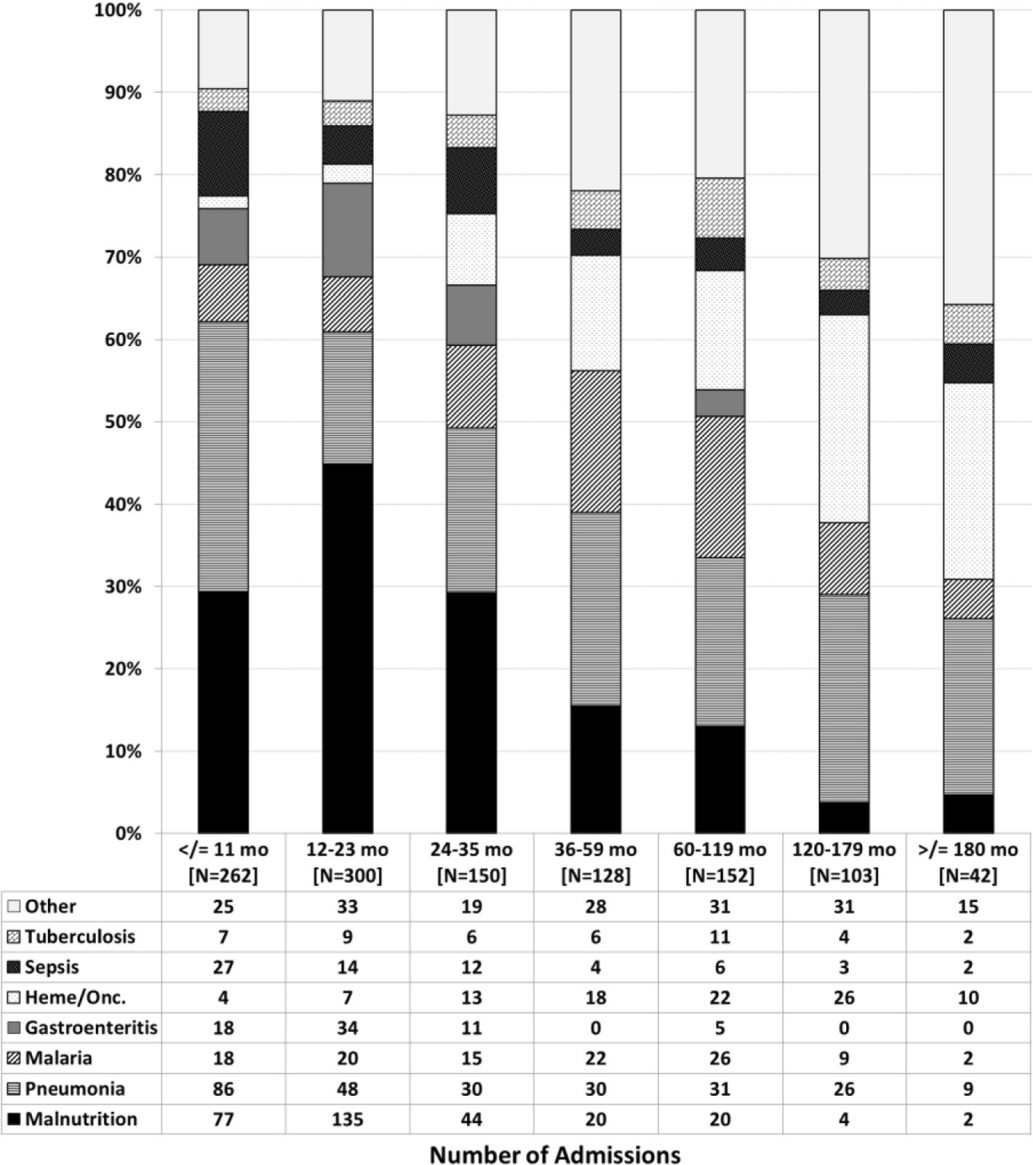
Stacked bar graph of primary admission diagnosis by age group, Malawi 2004–2010

**Fig. 2:**
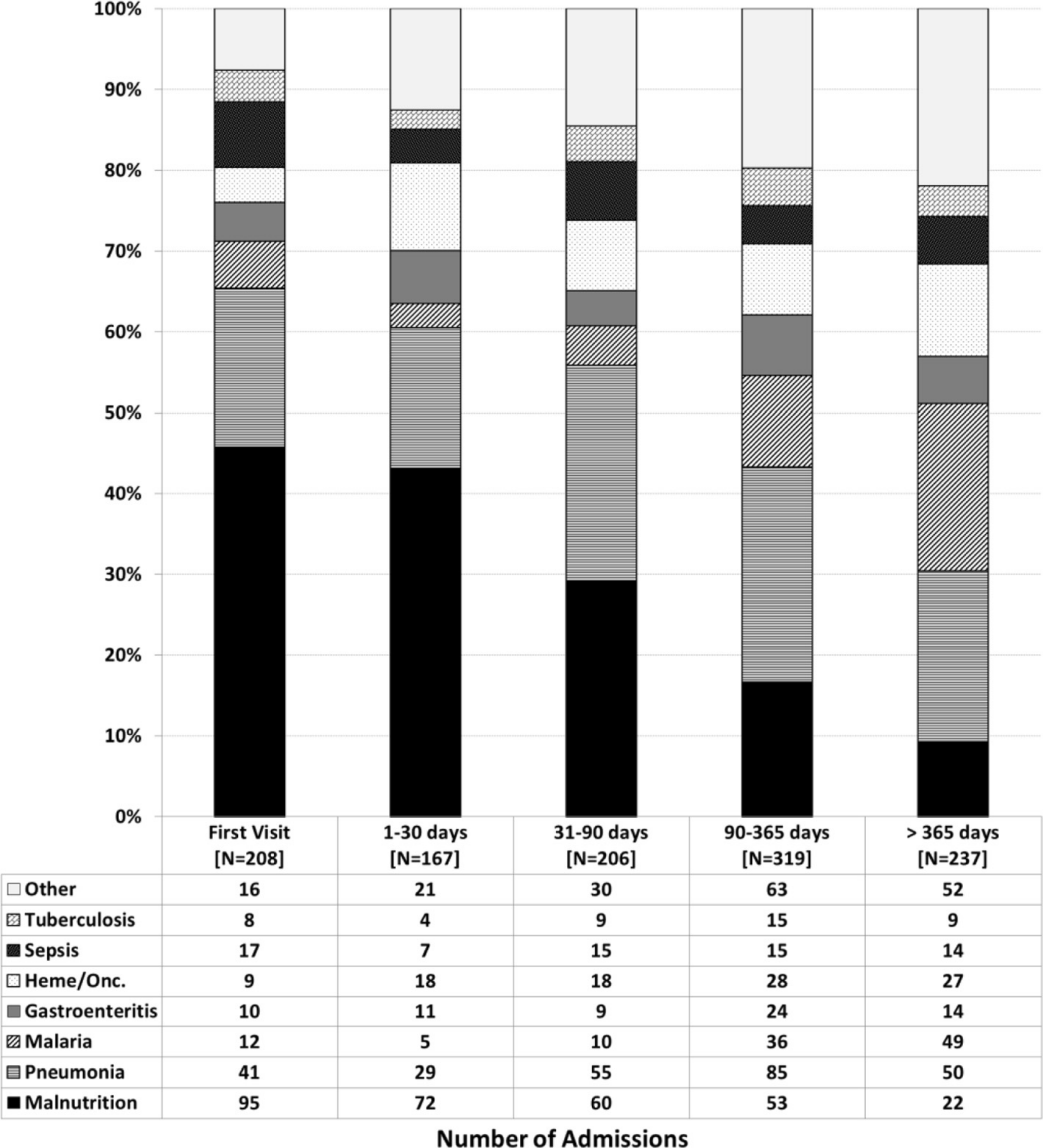
Stacked bar graph of primary admission diagnosis by time from clinic enrollment, Malawi 2004–2010

**Fig. 3:**
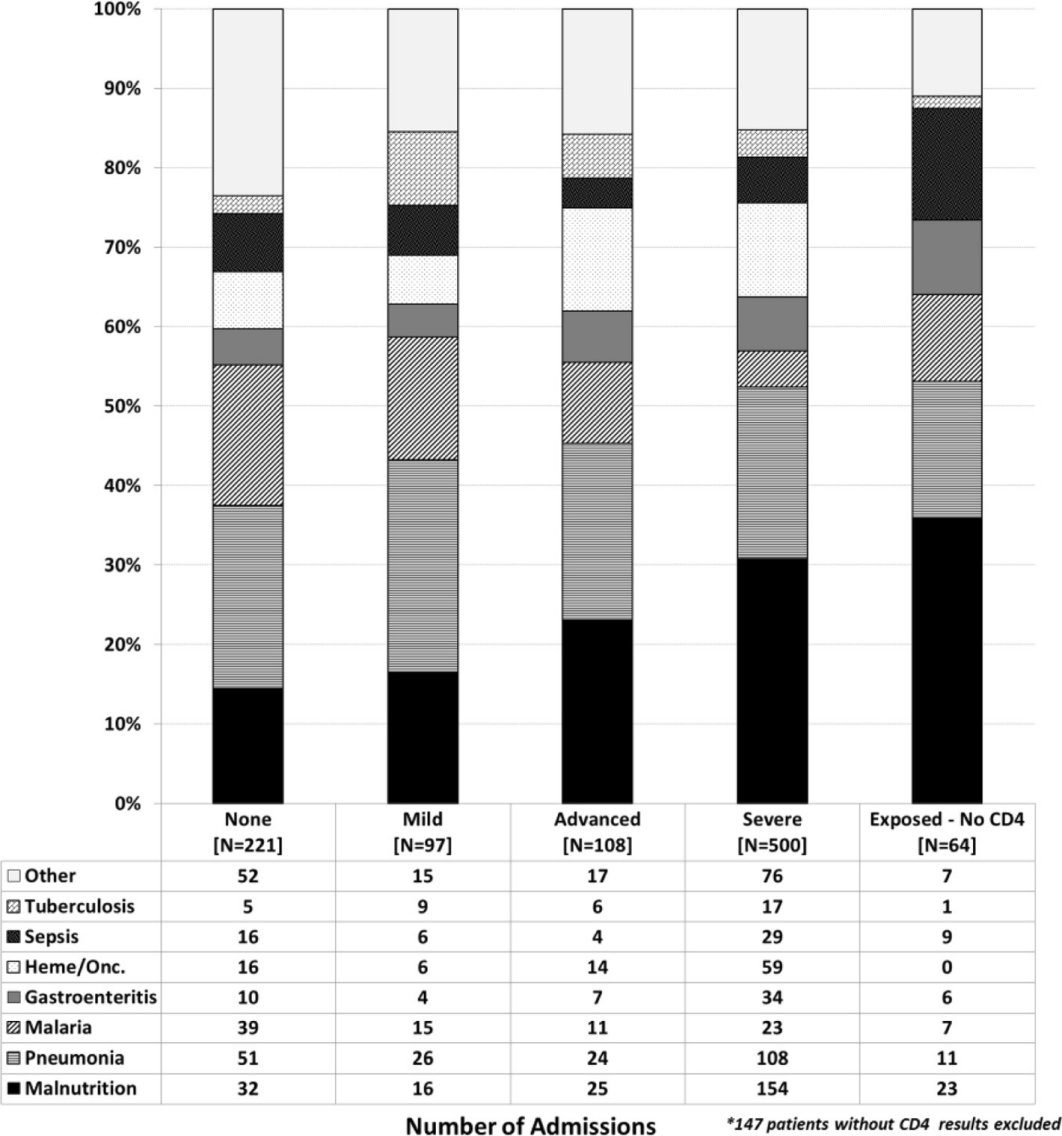
Stacked bar graph of primary admission diagnosis by immune suppression, Malawi 2004–2010

**Fig. 4:**
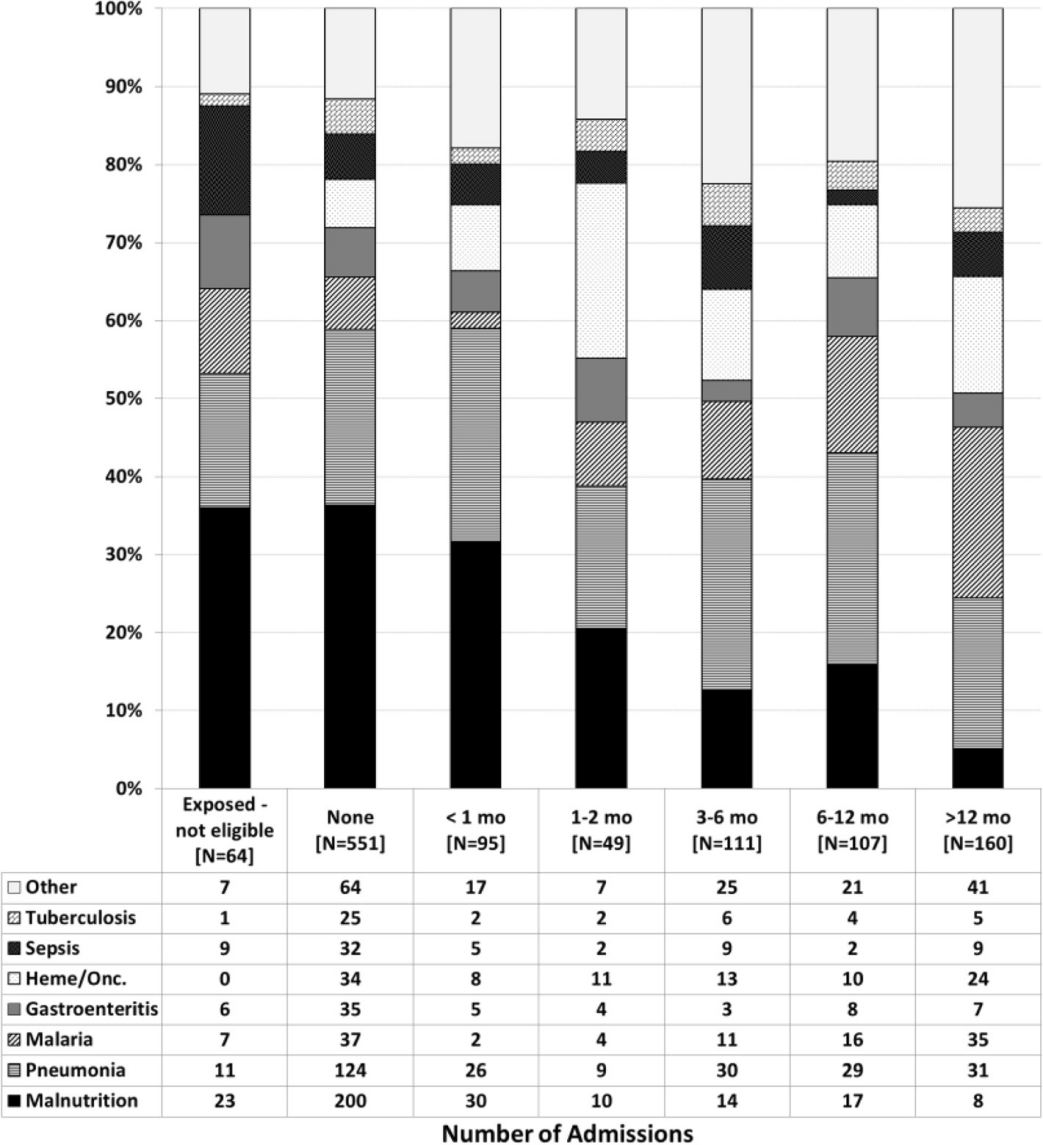
Stacked bar graph of primary admission diagnosis by time on ART, Malawi 2004–2010

The relative frequency of primary admission diagnoses differed based on various patient characteristics at each admission. Groupings based on age at admission were notable for decreasing frequency of malnutrition after 3 years of age, relatively stable frequency of pneumonia across age groups, and increasing frequency of oncologic admissions with increasing age (Fig. 1). Analysis based on the time interval between admission and enrollment in HIV care showed higher frequency of malnutrition early on, relatively stable frequency of pneumonia, and increasing frequency of malaria admissions with increased time in care (Fig. 2). Looking to the impact of immune status on admission diagnoses, malnutrition increased in frequency as a primary diagnosis with increasing immune suppression while the frequency of pneumonia showed less variation across immune categories (Fig. 3). The trends seen relative to the interval between ART initiation and hospitalization were similar to those seen for clinical enrollment with higher frequencies of malnutrition early on, relatively stable frequency of pneumonia, and increasing frequency of malaria admissions with increased time on ART (Fig. 4).

## Discussion

This study examines the inpatient admissions from a large cohort of HIV-infected and exposed children enrolled in outpatient HIV care. One of the most striking findings was the high mortality rates (30.8 %) noted in children who had been admitted. Previous research from the same clinic reported an overall mortality of 4.8 % in pediatric patients on ART [2]. While the cohorts in the studies were not the same, it is clear that patients requiring admission have high relative risk of mortality, highlighting the importance of both in-hospital and post-discharge care.

The clinic lost-to-follow up rate of 10.8 % for this cohort is similar to rates reported in pediatric ART populations from the region (8.4–11.5 %) [2, 17–19]. Patients admitted from the COE likely benefitted from the close physical proximity of the ward and outpatient site, as well as the shared resources of clinicians, labs, and support staff between the two, allowing for better coordination of inpatient and post-discharge care. In other settings where inpatient and outpatient care are provided in disparate locations by different providers, this linkage may be more problematic and lost-to-follow-up rates after discharge may be higher.

With respect to the demographics and characteristics of clinic patients who were admitted, the high proportion (49.4 %) of admissions in patients less than two years of age stands out. Previous studies have also shown hospitalizations in HIV-infected children are more common in this age group, and while these trends are similar to pediatric admission trends from this region regardless of HIV, the results stress the need for close monitoring of young children that are enrolled in HIV care [5, 9, 10, 20]. This also supports the need for expanded and improved early infant diagnosis (EID) programs to identify asymptomatic HIV-infected children and enroll them in care prior to disease progression which could require hospitalization.

The large percentage of admissions that occurred in patients who were only recently enrolled in HIV care (18.3 % of admissions occurred on the first day of enrollment into clinic and 33 % within the first month of entry into pediatric HIV care) and in those with evidence of severe immunosuppression (53.5 % had advanced or severe immunologic stage) reflects challenges with late entry into HIV care. These findings also support the need for robust EID programs as well as enhanced provider-initiated testing and counseling programs (PITC) to identify HIV-exposed and infected infants and children and speed their entry into care before they became symptomatic.

A large proportion of admissions occurred in confirmed HIV-infected patients who had not yet started ART, and over half (51.9 %) of these admissions occurred in children who had enrolled in care in the last 30 days. As discussed above, late entry to care certainly contributed to this trend with patients presenting with ART-eligible conditions requiring inpatient care at the time of diagnosis. While our data set did not allow for accurate determination of the proportion of these children who were actually ART-eligible given the timing of CD4 results and evolving national eligibility guidelines over the period of the study, we believe delays in initiation for ART-eligible children already in care also likely contributed to some of these admissions. Prior studies have demonstrated decreased pediatric hospital admission rates in patients who are started on ART [12, 21] and these results stress the importance of HIV clinic strategies that accelerate ART eligibility determinations, such as presumptive infant diagnosis, to avoid delays in definitive diagnosis with EID and timely access to CD4 testing. Recent guideline changes granting universal ART access to children under 5 years of age and all HIV-infected patients with TB should also help reduce the time needed to determine ART eligibility. Once eligibility is confirmed, clinic flows that prioritize and expedite pre-ART counseling and education for caregivers are critical.

The most common primary admission diagnosis was acute malnutrition (26.6 %.) It was the most common primary diagnosis in all children less than 3 years with a peak in the 12–23 month age group where it made up 45.0 % of admissions. It also made up the highest proportion of admissions that occurred within the first 3 months from enrollment into HIV care, with a decreasing proportion of admissions with increased time in care. Similarly, acute malnutrition also became a less likely cause of admission the longer a patient had been on ART. Finally, acute malnutrition was the most common cause of admission in exposed infants as well as in HIV-infected children with unknown, severe, and advanced immunologic stage, with a steadily increasing proportion of admissions with increasing immune suppression. These findings demonstrate the importance of routine nutritional screening and treatment as part of comprehensive HIV care and also reinforce the need for routine opt-out PITC, including criteria for presumptive HIV diagnosis, for children diagnosed with acute malnutrition in the sub-Saharan African region.

Pneumonia was the second most common overall primary admission, with the largest observed burden in patients less than 1 year of age. Presumed *pneumocystis jiroveci* pneumonia is made on clinical grounds in Malawi and was included in the all-cause pneumonia category, possibly explaining the relatively higher rates seen in younger patients, as they are known to be at higher risk for this condition [22]. Pneumonia represented a relatively stable proportion of primary admission diagnoses after the first year of life. The trends noted in acute malnutrition related to a time from enrollment in HIV care, immunosuppression, and time on ART were not observed with pneumonia. These results are consistent with data demonstrating the huge burden of pneumonia in all children in this setting, and support the argument that expanded access to pneumococcal (introduced in Malawi after the study period in 2011) and *Haemophilus influenza* type b (introduced in Malawi before the study period in 2002) immunization is urgently needed, particularly in at-risk HIV-infected children where the effectiveness of these vaccines in preventing invasive disease has been demonstrated [23–25].

Malaria was the third most common primary admission diagnosis, with the highest proportion of admissions noted in the 36–59 month age group (17.2 %). We hypothesize that in the period after protective maternal antibodies wane around 12 months, malaria is likely a steady cause of illness in this patient population and the observed frequencies in age groups younger than 5 years vary primarily because of the relative burden of other conditions, particularly acute malnutrition. The decreased relative frequency seen in the older groups could be related to acquired immunity from prior infections. The proportion of admissions due to malaria was noted to increase with longer time enrolled in care or on ART, and with improved immune status. This highlights the fact that healthy children with well-managed HIV are still vulnerable to endemic malaria in the region and comprehensive HIV care needs to include malaria education for caregivers and distribution of insecticide-treated bed nets.

This study has several strengths, most notably the large cohort of pediatric patients, the robust, standardized data available in the COE electronic medical records, and additional manual chart review to verify and augment information obtained from database queries. The principal limitation of our study methodology was the reliance on clinic charts alone, as it was not possible to retroactively link patients and trace records with the hospital archives. Some provisional diagnoses may have changed after admission, but unless they were documented in follow-up EMR notes, that information was not captured. In addition, the determination of primary admission diagnoses in patients with multiple problems was at times challenging. Finally, admissions to other hospitals or from other urgent care clinics may have been missed if not reported to COE clinicians and entered into the EMR.

The COE is a referral center for both the Northern and Central regions of the country for Kaposi’s sarcoma and other pediatric oncologic diseases, as well as for patients who required second line ART and other complicated cases. It also offers outpatient malnutrition and TB treatment for its HIV-infected and exposed patients, and the relative percentage of admissions due to these conditions might be higher than what would be seen in a standard high-volume national HIV site. Because some patients had multiple admissions, more chronic conditions such as Kaposi’s sarcoma may be over-represented in comparison to more acute illnesses such as malaria, pneumonia, and gastroenteritis which likely only required one hospitalization.

## Conclusion

The high burden of admissions in children less than 24 months and those newly enrolled in care suggests a need for continued improvement of early infant diagnosis and provider-initiated testing programs to link patients to care before they are symptomatic. Similarly, the high proportion of admissions in those not yet started on ART emphasizes the importance of rapid initiation of ART for eligible pediatric patients. These results reinforce the need for comprehensive care for HIV-infected and exposed children with special attention to nutrition. Strengthened programs for malaria prevention and introduction of pneumococcal vaccine are also needed as part of a comprehensive pediatric HIV care package. The demographics of children in HIV care will be changing as national PMTCT programs yield fewer infected infants and eligibility guidelines move towards universal pediatric ART access, which may result in different hospitalization patterns in the future.

## Abbreviations

ART: antiretroviral therapy
COE: center of excellence
EID: early infant diagnosis (of HIV)
EMR: electronic medical record
HIV: human immunodeficiency virus
IQR: interquartile range
KCH: Kamuzu Central Hospital
MUAC: mid-upper arm circumference
PITC: provider-initiated testing and counseling (for HIV)
PMTCT: prevention of mother to child transmission (of HIV)
TB: tuberculosis
WHO: World Health Organization
